# Potential radiosensitive germline biomarkers in the testes of wild mice after the Fukushima accident

**DOI:** 10.1002/2211-5463.13927

**Published:** 2024-12-02

**Authors:** Syun Tokita, Ryo Nakayama, Yohei Fujishima, Valerie Swee Ting Goh, Donovan Anderson, Ippei Uemura, Hikari Ikema, Jin Shibata, Yoh Kinoshita, Yoshinaka Shimizu, Hisashi Shinoda, Jun Goto, Maria Grazia Palmerini, Abdulla Mohamed Hatha, Takashi Satoh, Akifumi Nakata, Manabu Fukumoto, Tomisato Miura, Hideaki Yamashiro

**Affiliations:** ^1^ Graduate School of Science and Technology Niigata University Japan; ^2^ Department of Risk Analysis and Biodosimetry, Institute of Radiation Emergency Medicine Hirosaki University Aomori Japan; ^3^ Department of Radiobiology, Singapore Nuclear Research and Safety Initiative National University of Singapore Singapore; ^4^ Faculty of Pharmaceutical Sciences Hokkaido University of Science Sapporo Japan; ^5^ Graduate School of Dentistry Tohoku University Sendai Japan; ^6^ Institute for Research Administration Niigata University Japan; ^7^ Department of Life, Health and Environmental Sciences University of L'Aquila Italy; ^8^ Department of Marine Biology, Microbiology, Biochemistry Cochin University of Science and Technology India; ^9^ RIKEN Centre for Advanced Intelligence Project Pathology Informatics Team Tokyo Japan; ^10^ Field Centre for Sustainable Agriculture, Faculty of Agriculture Niigata University Japan

**Keywords:** *Apodemus speciosus*, Fukushima accident, low‐dose‐rate radiation, radiosensitive biomarkers

## Abstract

We investigated potential germline‐specific radiosensitive biomarkers in the testes of large Japanese field mice (*Apodemus speciosus*) exposed to low‐dose‐rate (LDR) radiation after the Fukushima accident. Fukushima wild mice testes were analysed via RNA‐sequencing to identify genes differentially expressed in the breeding and non‐breeding seasons when compared to controls. Results revealed significant changes during the breeding season, with *Lsp1* showing a considerable upregulation, while *Ptprk* and *Tspear* exhibited significant reductions. Conversely, in the non‐breeding season, *Fmo2* and *Fmo2* (highly similar) were significantly upregulated in radiation‐exposed Fukushima mice. qPCR analysis results were consistent with transcriptome sequencing, detecting *Lsp1* and *Ptprk* regulation in the testes of Fukushima mice. While differences in gene expression were observed, these do not imply any causal association between the identified biomarkers and chronic LDR exposure, as other factors such as the environment and developmental age may contribute. This study provides valuable insights into the reproductive biology is affected by environmental radiation and highlights the value of assessing the effects of chronic LDR radiation exposure on testicular health in wild mice.

## Graphical abstract

The study investigated radiosensitive biomarkers in the testes of Japanese field mice exposed to low‐dose (LDR) radiation after the Fukushima accident. Significant gene expression changes were observed during the breeding season, with *Lsp1* upregulation and *Ptprk* downregulating. These findings offer insights into how LDR radiation affects reproductive biology and could help assess chronic radiation's impact on testicular in wild mice.

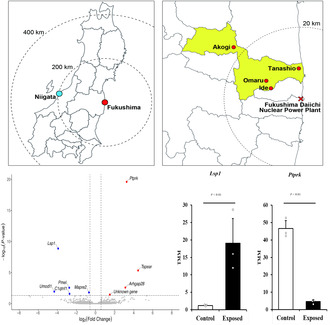

**Abbreviations**
DEG: differentially expressed gene
FDNPP: Fukushima Daiichi Nuclear Power Plant
FPKM: fragments per kilobase of transcript per million mapped reads
LDR: low‐dose‐rate
PCA: principal component analysis
qPCR: quantitative RT‐PCR
RNA‐seq: RNA‐sequencing
TMM: trimmed mean of M‐values
TPM: transcripts per million

The release of radioactive contaminants caused by the Fukushima Daiichi Nuclear Power Plant (FDNPP) accident had a significant impact on the environment and wildlife. In particular, the released radioactive ^137^Cs persist in the environment due to their long physical half‐life, which poses long‐term potential biological concerns. Radiation monitoring surveys of wildlife within the ex‐evacuation zone, a 20‐km radius region surrounding FDNPP, have been conducted, focusing on species such as large Japanese field mice [1, 2], Japanese macaques [3, 4, 5], wild boars [6, 7, 8, 9], and raccoons [10]. Characterising the biological effects on wildlife is crucial for understanding the impact of chronic low‐dose‐rate (LDR) radiation exposure on humans. The current global threat of nuclear terrorism and radiation safety risks highlight the need for further radiation monitoring and research on the biological effects on wildlife after the FDNPP accident.

Over the past few decades, radiobiologists have shown a keen interest in studying the biological effects of chronic LDR radiation exposure on germ cells. Studies have reported that the exposure of male germ cells to chronic LDR radiation induces hormesis and adaptive responses in terms of apoptosis, whereas exposure to high‐dose‐rate radiation inhibits metabolism, antioxidant capacity, proliferation, and maturation [11]. Until recently, the effects of radiation that were primarily considered included nuclear DNA damage and the subsequent repair mechanisms. However, recent molecular biological studies have revealed that the effects of ionising radiation exposure are highly dependent on the activation and regulation of molecular factors which influence cell survival and proliferation [12]. In controlled laboratory mice experiments with gamma ray irradiation, 6.0 mGy·h^−1^ LDR radiation exposure affected the fertility‐related DNA methylation pattern [13]. Furthermore, 3.4 mGy·h^−1^ LDR radiation exposure for 100 days was reported to decrease the number of spermatogonia to a greater extent than 0.85 Gy·min^−1^ high‐dose‐rate radiation (51 Gy·h^−1^, 2 Gy × 4 times at 1‐week intervals) [14]. In contrast, another report suggested that 0.70 mGy·h^−1^ did not induce greater sperm abnormalities compared to HDR exposure, proposing that damaged spermatogonia were effectively repaired following LDR irradiation. While radiation at 0.70 mGy·h^−1^ or less may not induce DNA damage, it was sufficiently harmful to induce spermatogonia cell death [15]. These results suggested that a threshold in dose rate or the total dose of LDR radiation exposure may exist with regard to spermatogenesis and reproductive function. We hypothesised that it is imperative to prioritise the self‐renewal and differentiation of spermatogonia stem cells, meticulously analysing the intricate dynamics of early spermatogenesis and its enduring impact on radiosensitivity.

Previous studies on the matter have explored the dynamics and functions of reproductive sperm cells following chronic LDR radiation exposure in mice. Okano *et al*. [16] did not observe any effect on spermatogenesis in the testes of large Japanese field mice based on the analysis of apoptosis and sperm abnormalities. Nihei *et al*. [17] demonstrated that sperm fertilisation capacity in large Japanese field mice was not affected by 10 years of cryopreservation, indicating that both chronic LDR radiation exposure and cryopreservation do not affect male fertility. Takino *et al*. [18] suggested that chronic LDR radiation exposure promotes germ cell proliferation in the testes of large Japanese field mice. However, the effects of chronic LDR radiation exposure on gene expression in spermatogenic cells have not yet been elucidated.

In this study, we performed transcriptomic analysis to characterise gene expression in spermatogenic cells following chronic LDR radiation and thus determine germline‐specific radiosensitivity biomarkers in the testes of large Japanese field mice exposed to chronic LDR radiation after the FDNPP accident.

## Materials and methods

### Collection of large Japanese field mice (*Apodemus speciosus*)

All procedures were conducted in compliance with the guidelines of the Ethics Committee for the Care and Use of Laboratory Animals for Research of Niigata University, Japan (Protocol No. 26‐80‐2).

**Fig. 1:**
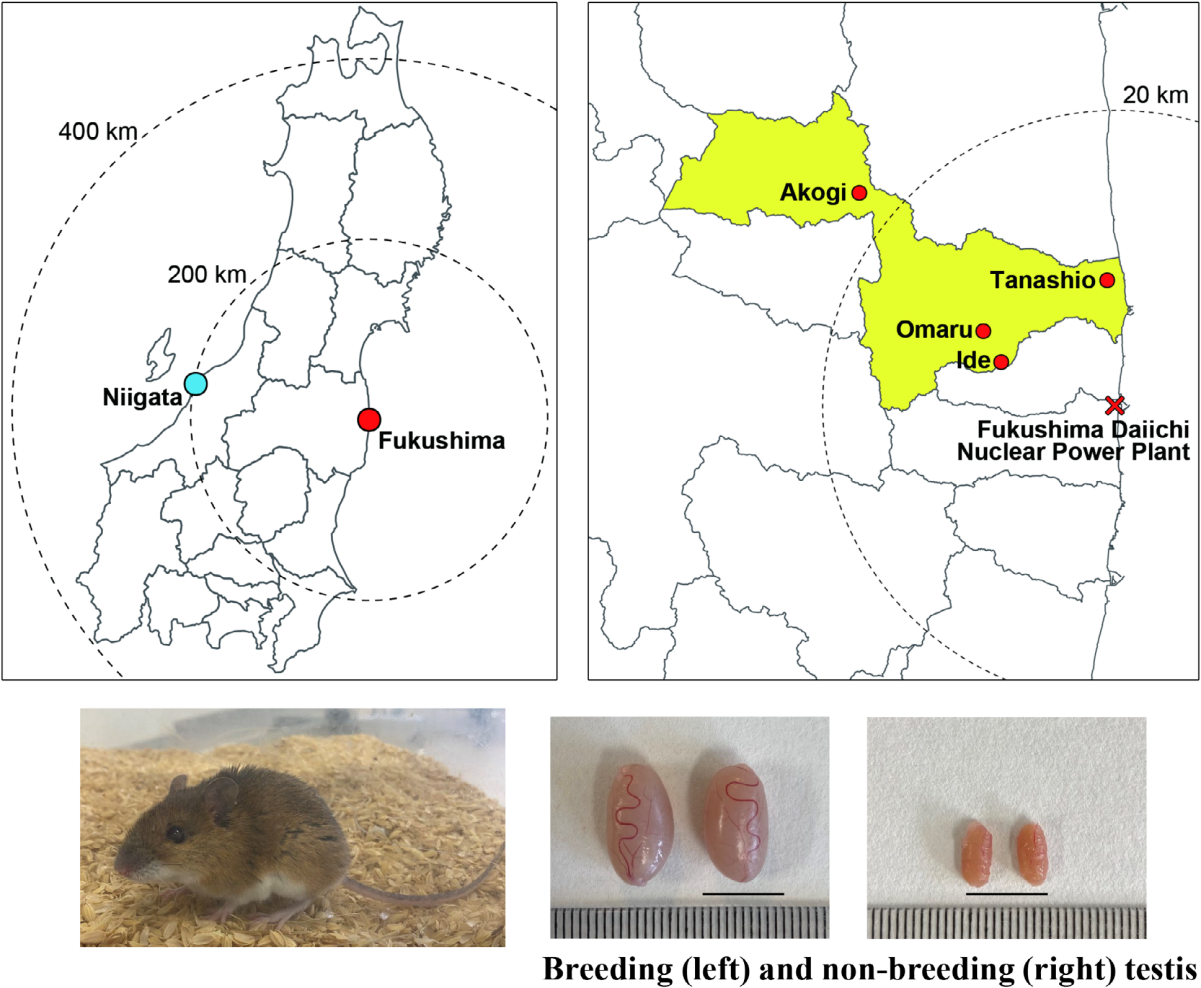
Sampling locations included a control site in Niigata Prefecture, Japan, as well as three areas in Fukushima Prefecture, Japan, that were affected by radioactive contamination. Testes were sampled from large Japanese field mice at all locations, during both the breeding and non‐breeding seasons. The blue circle indicates a large Japanese field mice sampling point in Niigata. The red circles indicate sampling points in areas that were considered evacuation zones. Spatial data were obtained from the National Land Numerical Information download service (http://nlftp.mlit.go.jp/ksj/index.html). Scale bar = 10 mm.

Contaminated mice were captured in Namie Town, Fukushima Prefecture, while control mice were captured in Niigata City, Niigata Prefecture, in Japan (Fig. 1). Large Japanese field mice were captured using baited Sherman‐type live traps (H. B. Sherman Traps, Inc., Tallahassee, FL, USA). Sampling in Namie Town was conducted from November 2012 to November 2013, when the area was designated as the evacuation zone following the FDNPP accident. Sampling at Kakuta Mountain in Niigata City was conducted from July 2020 to October 2020. The 3Rs principle (reduction, refinement, and replacement) in animal experiments was followed such that a minimal number of captured wild mice was used. Table 1 shows information related to each of the mice sampled at each site and the ambient dose rates measured. Sample Fu_6 from the contaminated location of Tanashiro had much lower dosimetry than other samples from Fukushima.

**Table 1:** Individual information for large Japanese field mice. A, applicable; N/A, not applicable.

Group	ID	Site	Sampling date	Body weight (g)	Ambient dose‐rate (μSv·h^−1^)	Average diameter of seminiferous tubule (μm)	Presence of sperm
Niigata (Control)	Ni_1	Kakuta	22 Jul. 2020	46.3	0.0597^a^	179	—
	Ni_2		4 Aug. 2020	52.2		179	—
	Ni_3		20 Aug. 2020	48.9		193	—
	Ni_4		14 Oct. 2020	39.8		58	—
	Ni_5		14 Oct. 2020	44.3		63	—
	Ni_6		14 Oct. 2020	44.3		90	—
Fukushima	Fu_1	Ide	6 Nov. 2012	24.5	24.5	—	A
	Fu_2	Akogi	19 Apr. 2013	38.7	15.2	—	A
	Fu_3	Akogi	19 Apr. 2013	32.4	15.2	—	A
	Fu_4	Akogi	6 Nov. 2012	23.5	24.2	—	N/A
	Fu_5	Ide	6 Nov. 2012	32.9	23.7	—	N/A
	Fu_6	Tanashio	7 Nov. 2012	30.3	0.52	—	N/A

^a^ Outdoor ambient gamma dose rates and doses from external exposure to terrestrial radiation in Niigata, Japan; Omori *et al*. [44].

### Tissue sampling and processing

The wild mice were euthanasia and sacrificed by cervical dislocation and both testes were excised. For RNA‐sequencing (RNA‐seq), testes were frozen in liquid nitrogen just after capture and then stored at −80 °C until analysis. Testes in the breeding season were identified based on the presence of sperm and/or epididymal duct diameter [19, 20].

### Measurement of ambient dose rates at sampling sites

Ambient dose rates (μSv·h^−1^) were measured using a NaI (Tl) scintillation survey meter (TCS‐171B, ALOKA Co., Tokyo, Japan) at 1 m above the ground surface at each sampling site.

### RNA‐seq

We performed RNA‐seq using testes from 12 mice classified based on the presence of sperm or the epididymal duct diameter. Testes from three mice during the breeding season and three mice during the non‐breeding season were compared in both the Fukushima and the control groups. Total RNA was extracted using the RNeasy Mini Kit (Qiagen, Hilden, Germany). The integrity and nucleic acid concentration of total RNA were assessed using an Agilent 2100 Bioanalyzer (Agilent Technologies, Palo Alto, CA, USA) for quality evaluation. Using the NEBNext Poly(A) mRNA Magnetic Isolation Module (New England Biolabs, Ipswich, MA, USA), mRNA was purified from 400 ng of total RNA. Sequencing libraries were prepared using the NEBNext Ultra II Directional RNA Library Prep Kit (New England Biolabs), followed by sequencing on a NovaSeq6000 platform (Illumina, CA, USA) with 150 bp paired‐end reads.

### Analysis of differentially expressed genes (DEGs)

The obtained sequence reads were subjected to a quality inspection using fastqc (Version 0.11.7) [21]. Trimming was then conducted using trimmomatic (Version 0.38) [22] to eliminate low‐quality reads, adapter sequences, and short reads. The trimmed reads were mapped to the reference genome using bowtie2 (Version 2.3.4.2) [23] based on the transcriptome data assembled through *de novo* analysis. Gene‐specific read counts, transcripts per million (TPM), and fragments per kilobase of transcript per million mapped reads (FPKM) were calculated using trinity (Version 2.13.1) [24, 25]. Thereafter, the trimmed mean of M‐values (TMM) normalisation was performed using sample read counts. Hierarchical clustering, principal component analysis (PCA), and correlation evaluation between samples based on TPM and TMM values were conducted on the genes detected using the stats package. DEGs were identified as genes with at least a two‐fold change in expression and a false discovery rate of less than 0.05 during the breeding and non‐breeding seasons.

### Measurement of mRNA levels via quantitative RT‐PCR (qPCR)

Total RNA from testes was extracted using ISOGEN (Nippon Gene Co., Ltd., Tokyo, Japan). cDNA was prepared from 1 μg total RNA via reverse transcription using ReverTra Ace (Toyobo, Osaka, Japan). qPCR was performed with a KAPA SYBR Fast qPCR Kit (NIPPON Genetics Co., Ltd., Tokyo, Japan). Quantitative RT‐PCR analysis was performed using QuantStudio 3 Real‐Time PCR System (Applied Biosystems, Foster City, CA, USA). Primers used to examine expression levels are listed in Table 2. Table 3 shows individual information for large Japanese field mice.

**Table 2:** Primers used in this study. Actb, β‐actin; *Fmo2 HS*, flavin‐containing monooxygenase 2 highly similar; *Fmo2*, flavin‐containing monooxygenase 2; *Lsp1*, lymphocyte specific 1; *Ptprk*, protein tyrosine phosphatase receptor type K; *Tspear*, thrombospondin type laminin G domain and EAR repeats.

Gene	Sequence (5′–3′)
Actb	
Fw	GATGTGGATCAGCAAGCAGGA
Rev	AAACGCAGCTCAGTAACAGTCC
*Lsp1*	
Fw	ATGGCCGTAGCCAGTACCAA
Rev	GGGGTGCTCTTGATGGTGGA
*Ptprk*	
Fw	GGCAGCCAGCAGCTTTCATC
Rev	CAGCCCTGAGACAGGTCCAC
*Tspear*	
Fw	AACCACCGAGAAGGGGACAA
Rev	CACCTGGGTGGAAGTGCCAT
*Fmo2*	
Fw	GCGCTAGCATCTACCGCTCT
Rev	CTCCGGCATCGGGAAGTCAC
*Fmo2 HS*	
Fw	TTCAAAGGCTTGTGTAGCTTGC
Rev	GTCTGCAGTATCTGACTCTGGC

**Table 3:** Individual sampling information for each large Japanese field mice.

Group	ID	Site	Sampling date	Body weight (g)	Ambient dose‐rate (μSv·h^−1^)
Fukushima	191	Tanashio	5 Nov. 2012	25.7	0.52
	222	Ide	6 Nov. 2012	40.5	23.7
	230	Tanashio	7 Nov. 2012	31.6	0.52
	257	Ide	19 Mar. 2013	30.2	15.8
	258	Ide	19 Mar. 2013	28.4	15.8
	259	Akogi	19 Mar. 2013	38.7	15.1
	260	Akogi	19 Mar. 2013	32.4	15.1
	372	Akogi	10 Mar. 2014	47.6	5.6
	389	Tanashio	10 Mar. 2014	39	0.26
	390	Tanashio	10 Mar. 2014	49	0.26
	398	Akougi	10 Mar. 2014	36.8	5.6
	400	Tanashio	10 Mar. 2014	47.2	0.26
	797	Omaru	22 Nov. 2019	39.1	9.1
	842	Omaru	21 May 2022	48.9	7.83
	848	Omaru	15 Oct. 2021	49.3	7.36

### Statistical analysis

Statistical analysis was performed using edger (version 3.42.4) [26] using TMM‐normalised values. The extracted DEGs were z‐scored using TMM‐normalised values, and a heatmap was generated. Volcano plots were used for an overview of the gene expression fold changes and their statistical significance, generated using r 4.3.2 [27], with the graphic front‐end rstudio version 2023.12.0 + 369 [28].

Statistical analyses of qPCR data were performed using the excel statistical software package (Bell Curve for Excel v.3.20, Social Survey Research Information Co., Ltd., Tokyo, Japan). Statistically significant differences were evaluated using Dunnett's test. In Dunnett's test, the values of the Niigata were used as a control. Data are presented as the mean ± SD. Statistical significance was defined as *P* < 0.05 or 0.01.

## Results

### Gene expression profiles of testes in the Fukushima and control groups

**Fig. 2:**
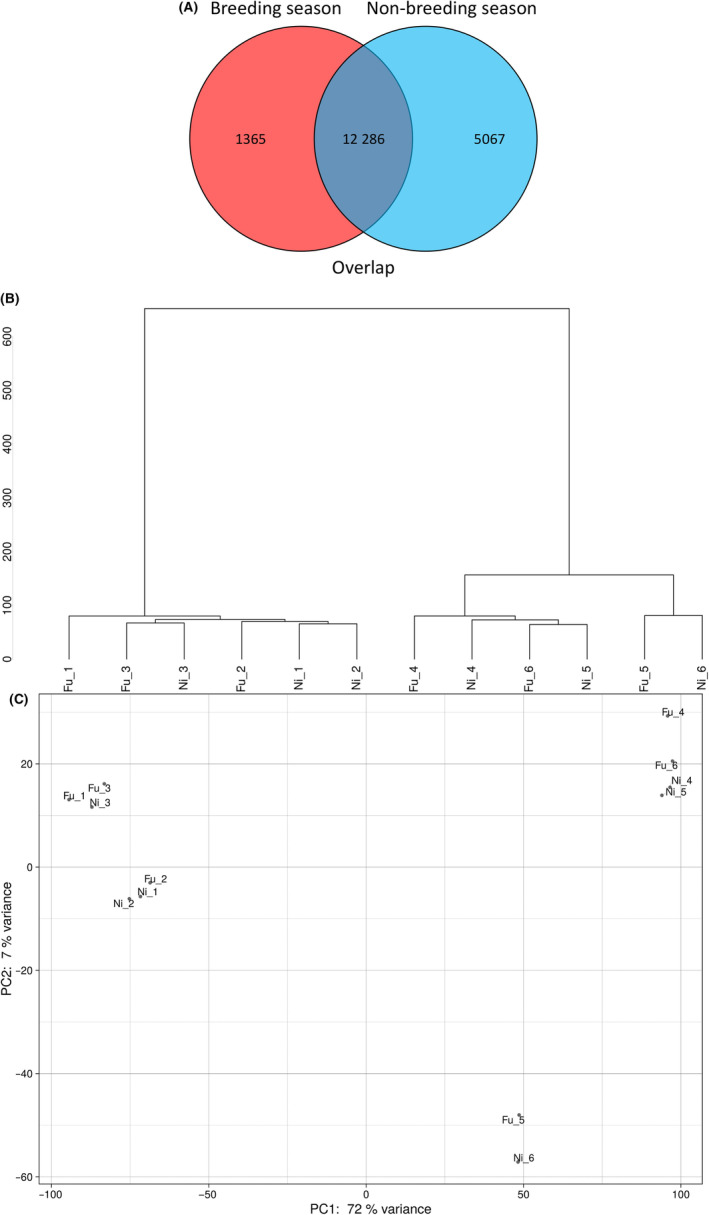
DEGs between in the testis of large Japanese field mice in the breeding versus non‐breeding season. (A) Venn diagram of gene expression. The figure shows genes expressed only in the breeding season (red circle) and the non‐breeding (blue circle), with the overlapping part of the circle representing genes overlapping between the groups. The degree of similarity in gene expression between samples was visualised in hierarchical clustering (B) and principal component analysis (C).

Contaminated and controlled large Japanese field mice in both breeding and non‐breeding seasons were sampled from the ex‐evacuation zone of the FDNPP accident and in Niigata, respectively (Fig. 1). Significant differences in gene expression profiles between the Fukushima and the control groups were recorded. During the breeding season, when compared to the control group, 13 651 genes were either upregulated or downregulated in the Fukushima group (Tables S1 and S2; Fig. 2A). In the non‐breeding season, a total of 17 353 genes were differentially regulated in the Fukushima group as compared to the control group. A Venn diagram of DEGs identified 12 286 key genes at the crossover points.

### Radiation‐sensitive genes in the testes following chronic LDR radiation exposure

To identify radiation‐sensitive genes in the testes of large Japanese field mice and to elucidate the effect of chronic LDR radiation exposure on testicular gene expression, RNA‐seq was conducted, followed by DEG analysis with hierarchical clustering, PCA, correlation, and heatmap visualisation.

**Fig. 3:**
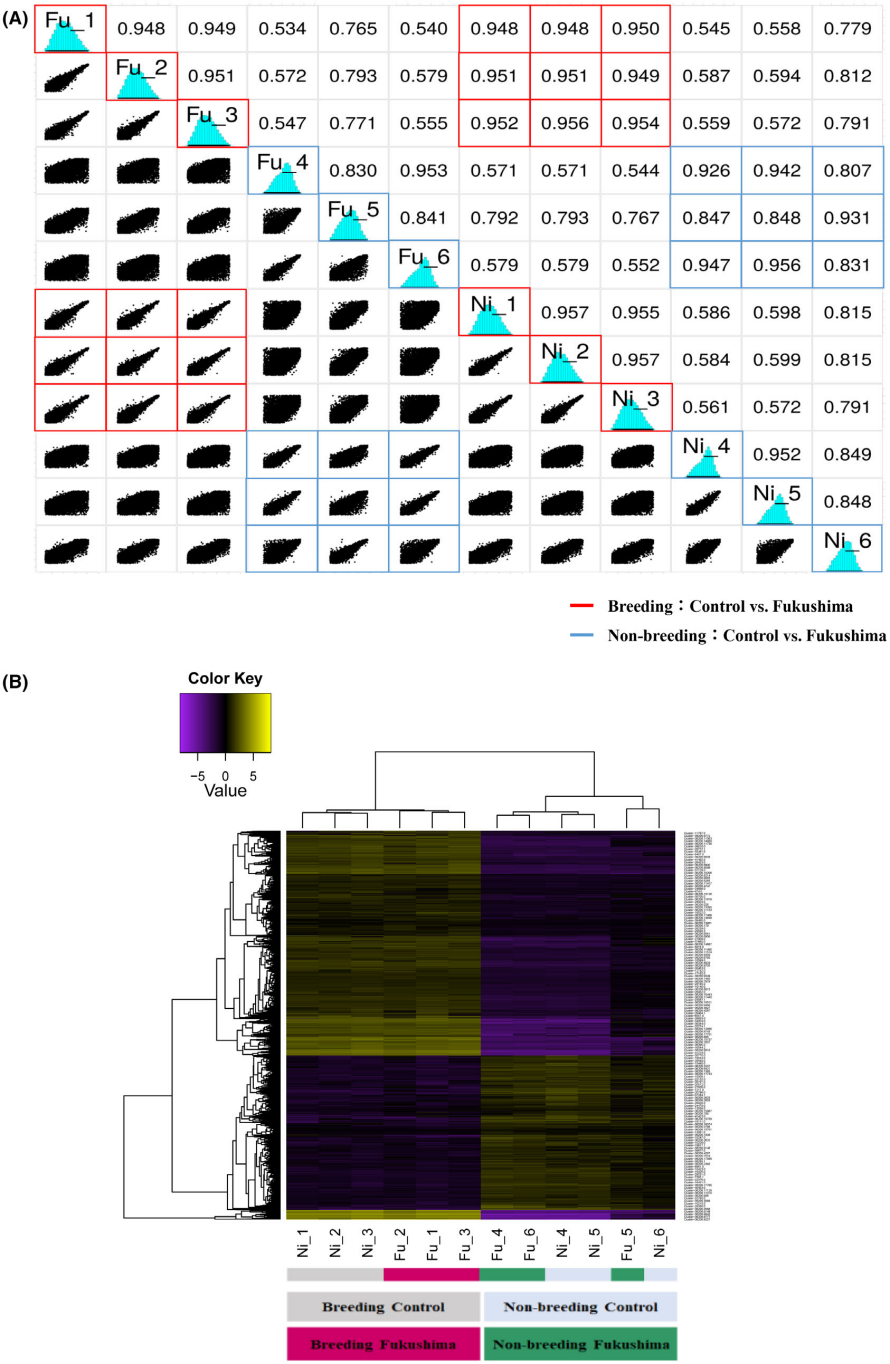
Pairwise scatter plot and testicular gene expression heatmap comparing the testes sampled from contaminated and control groups. (A) Pairwise scatter plot of DEGs between Fukushima and control groups. (B) Heatmap of DEGs. Yellow represents high‐expression genes, and purple represents low‐expression genes.

In Fig. 2B,C, hierarchical clustering and PCA based on DEGs showed that samples from both Fukushima and Niigata broadly classified into breeding and non‐breeding seasons, with the individual Fu_5 and Ni_6 clustering separately. Figure 3A shows a pairwise scatter plot of DEGs between Fukushima and Niigata mice. Similarities in gene expression were also observed between during breeding and non‐breeding seasons in both Fukushima and Niigata mice. The heatmap in Fig. 3B shows the overall expression trends for each sample (column) and gene (row). Each cell corresponds to the expression level in the respective sample or gene (exon). Yellow indicates higher expression levels, whereas purple indicates lower expression levels. The heatmap based on RNA‐seq results revealed expression differences between the breeding and non‐breeding groups of both Fukushima and Niigata mice, showing coordinated expression patterns specific to the breeding season.

**Fig. 4:**
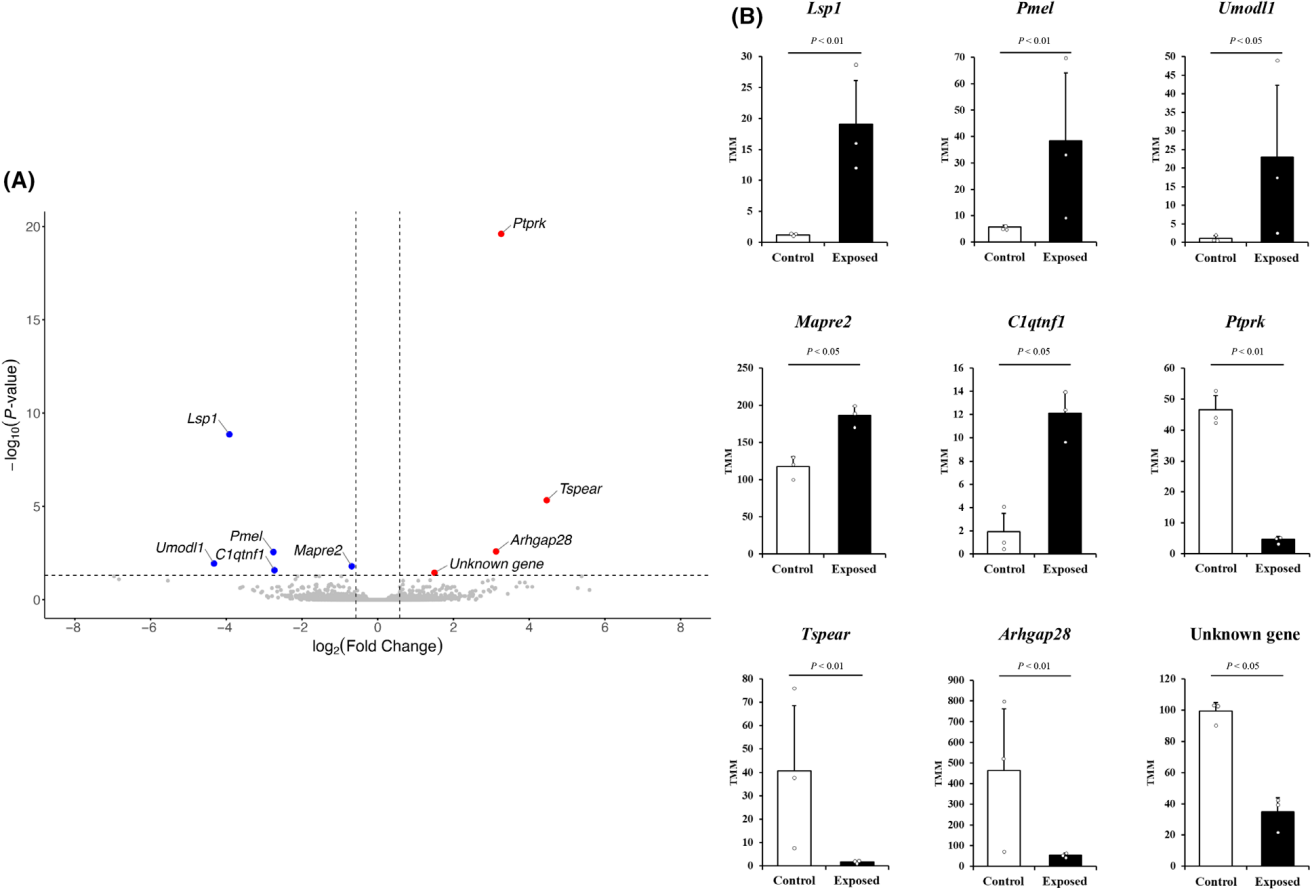
Significant gene expression changes in the testes of contaminated and control mice sampled during the breeding season. (A) Volcano plot of differential gene expression patterns. (B) Bar plots of significantly altered gene expression ratio in the testes. No. of wild mice used for breeding season testes of contaminated (*n* = 3) and control (*n* = 3). All data are expressed in relative units. Statistical analysis was performed using edger (Version 3.42.4) using TMM‐normalised values. Data are presented as mean ± SE. *Arhgap28*, rho GTPase‐activating protein 28; *C1qtnf1*, C1q and tumour necrosis factor‐related protein 1; *Lsp1*, lymphocyte specific 1; *Mapre2*, microtubule‐associated protein RP/EB family member 2; *Pmel*, premelanosome protein; *Ptprk*, protein tyrosine phosphatase receptor type K; RP23‐220C16, *Mus musculus* BAC clone RP23‐220C16 from 8; *Tspear*, thrombospondin type laminin G domain and EAR repeats; *Umodl1*, uromodulin‐like 1.

DEGs between the contaminated and control groups were separately identified in breeding and non‐breeding seasons. In the volcano plot comparing control and contaminated mice in the breeding season (Fig. 4A), nine DEGs were detected in the testes, including lymphocyte‐specific 1 (*Lsp1*), protein tyrosine phosphatase receptor type K (*Ptprk*), thrombospondin type laminin G domain and EAR repeats (*Tspear*), Rho GTPase‐activating protein 28 (*Arhgap28*), premelanosome protein (*Pmel*), uromodulin‐like 1 (*Umodl1*), microtubule‐associated protein RP/EB family member 2 (*Mapre2*), C1q, and tumour necrosis factor‐related protein 1 (*C1qtnf1*), and an unknown gene (RP23‐220C16). In particular, the mRNA expression levels of *Lsp1*, *Ptprk*, and *Tspear* in the testes of the contaminated group were significantly different (*P* < 0.05) from those in the control group. Furthermore, the mRNA expression levels of *Arhgap28*, *Pmel*, *Umodl1*, *Mapre2*, *C1qtnf1*, and an unknown gene (RP23‐220C16) in the exposed group also exhibited a significant difference (*P* < 0.05) compared to those in the control group (Fig. 4B).

**Fig. 5:**
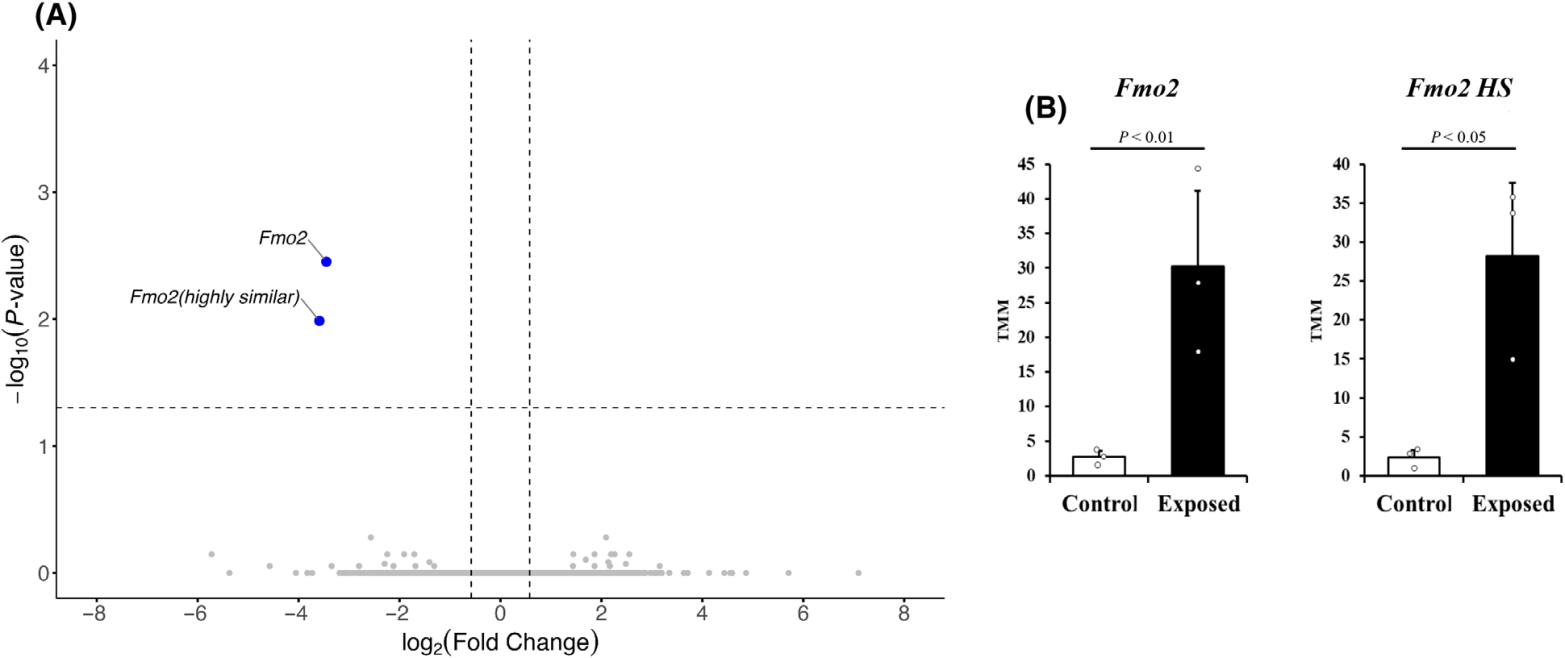
Significant gene expression changes in the non‐breeding season testes of contaminated and control mice. (A) Volcano plot of the differential gene expression patterns. (B) Bar plots of significantly altered gene expression ratio in the testes. No. of wild mice used for non‐breeding season testes of contaminated (*n* = 3) and control (*n* = 3). All data are expressed in relative units. Statistical analysis was performed using edger (Version 3.42.4) using TMM‐normalised values. Data are presented as mean ± SE. *Fmo2 HS*, flavin‐containing monooxygenase 2 highly similar; *Fmo2*, flavin‐containing monooxygenase 2.

Flavin‐containing monooxygenase 2 (*Fmo2*) and its highly similar (*Fmo2 HS*) were identified as DEGs in the non‐breeding season testes (Fig. 5A). Their mRNA expression levels showed a significant increase (*P* < 0.05) compared to those in the control group (Fig. 5B).

### Validation of transcriptomic data

We evaluated the mRNA expression levels of the five significant DEGs, *Lsp1*, *Ptprk*, *Tspear*, *Fmo2*, and *Fmo2 HS*, to confirm DEGs discovered via RNA‐seq analysis. These genes play a role in the regulation of phosphoprotein phosphatase activity, G protein‐coupled receptor activity, oxidation regulation process, and GTP activator activity, in addition to being closely associated with spermatogonia stem cell activity during spermatogenesis (Table S1 and Table 2).

**Fig. 6:**
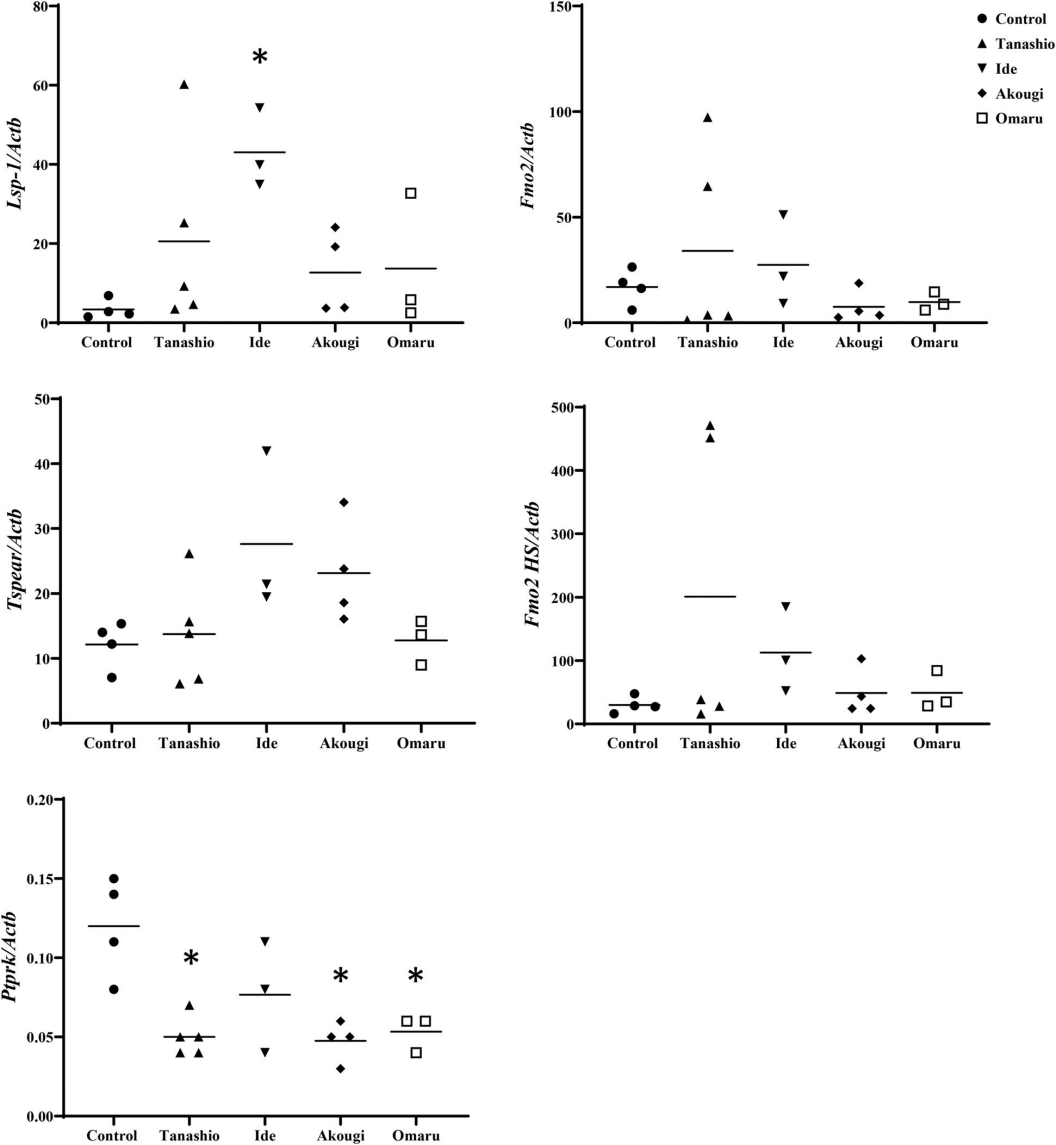
qPCR detection of potential biomarkers *Lsp1*, *Ptprk*, *Tspear*, *Fmo2*, and *Fmo2 HS* in the testis of large Japanese field mice. *Fmo2 HS*, flavin‐containing monooxygenase 2 highly similar; *Fmo2*, flavin‐containing monooxygenase 2; *Lsp1*, lymphocyte specific 1; *Ptprk*, protein tyrosine phosphatase receptor type K; *Tspear*, thrombospondin type laminin G domain and EAR repeats. No. of wild mice used for Niigata (*n* = 4), Tanashio (*n* = 5), Ide (*n* = 3), Akogi (*n* = 4), and Omaru (*n* = 3). Statistically significant differences were evaluated using Dunnett's test. *represents the significance of difference (*P* < 0.05).

qPCR results revealed significant changes during the breeding season in contaminated Fukushima mice, with *Lsp1* showing a significant (*P* < 0.05) increase in mRNA expression, whereas *Ptprk* exhibited significant (*P* < 0.05) reductions (Fig. 6). *Tspear*, *Fmo2*, and *Fmo2 HS* were not significantly expressed in contaminated Fukushima mice.

## Discussion

The long‐term radiation contamination caused by nuclear accidents raises significant concerns regarding the biological consequences of radiation. In this context, wild mice from the ex‐evacuation area in Fukushima prefecture serve as excellent indicator species owing to their continuous exposure to radiation over an extended period. By elucidating the environmentally responsive dynamics of spermatogenic cells in the testes of contaminated large Japanese field mice, we can better understand the effects of chronic LDR radiation on reproductive cells in both wild animals and humans.

In this study, we investigated the impact of chronic LDR radiation exposure resulting from the FDNPP accident on germline‐specific gene expression in the testes of male large Japanese field mice collected from the ex‐evacuation zone of the accident. The ambient dose rate measured across all our sampling sites ranged from 0.52 to 24.5 μSv·h^−1^. Kubota *et al*. [29] estimated that these ambient dose rates were equivalent to an external radiation dose‐rate of approximately 0.01 to 0.59 mGy·day^−1^ for the mice. According to the International Commission on Radiological Protection (ICRP), the reference band level for environmental exposure of 0.1 to 1 mGy·day^−1^ is associated with a ‘very low probability of radioactive effects that could result in reduced reproductive success’ in rats genetically similar to Japanese field large mice. At higher doses of 1–10 mGy·day^−1^, there is a potential for reduced reproductive success due to decreased fertility in both males and females [30, 31, 32]. Given that the highest external radiation dose rate measured in our study was 0.59 mGy·day^−1^ in November 2012, it is unlikely that large Japanese field mice exposed to chronic LDR radiation in the ex‐evacuation zone would experience fertility issues. However, internal concentrations of radionuclides were not measured in our study, and our ambient dose measurements were taken at 1 m above the ground. Internal radionuclide accumulation is possible, particularly if significant amounts of ^137^Cs and ^90^Sr are bioaccumulated, which could contribute more to absorbed dose rates in mice. Additionally, if ambient dose rates were measured closer to the forest floor or in burrows, external exposure could be higher [33]. In such cases, the mice sampled in our most contaminated area might have exceeded the ICRP's low‐probability reference band level of 1 mGy·day^−1^. Furthermore, ambient dose rates were measured at specific sampling sites 1 day before sample collection, which may not be reflective of the entire period of exposure. Distance travelled by the mice in their areas of habitation should also be considered to account for the heterogeneous ambient dose rates in different areas of the ex‐evacuation zone. Although the ambient dose rates measured in the contaminated sampling sites could only serve as a very rough estimate of chronic LDR exposure, there should still be a difference in accumulated dose between mice in Fukushima and Niigata.

We previously reported several findings in large Japanese field mice assessing the influence of chronic LDR radiation exposure associated with the FDNPP accident on male reproductivity. Ohdaira *et al*. [34] used histological analyses to show normal spermatogenesis in large Japanese field mice caught in heavily contaminated areas during the breeding season. Electron probe X‐ray microanalysis did not detect radioactive caesium in the testes. Nihei *et al*. [17] showed that chronic LDR radiation exposure did not affect the *in vitro* fertilisation capacity of frozen–thawed cryopreserved sperm in large Japanese field mice. Furthermore, Takino *et al*. [18] showed a tendency for enhanced spermatogenesis in large Japanese field mice living in areas affected by the FDNPP accident, although the molecular mechanisms linked to enhanced spermatogenesis remain unclear. To complement our previous analyses, we decided to identify DEGs in contaminated and control mice from breeding and non‐breeding seasons. These DEGs could serve as potential biomarkers and further our understanding of the effects of chronic LDR on spermatogenesis and reproduction.

A total of 17 353 genes were differentially regulated in the contaminated group as compared to the control group, with 12 286 DEGs overlapping. We also sought to perform Gene Ontology analysis on DEGs; however, there were a limited number of DEGs and a lack of annotated GO terms (results not shown). In hierarchical clustering and PCA of the DEGs identified, most of the samples clustered separately in the breeding and non‐breeding seasons, whereas Fu_5 and Ni_6 clustered together. In the correlation and heatmap analysis, both contaminated and control mice were instead clustered in two distinct groups of breeding and non‐breeding seasons. These differences might be due to the reproductive period affecting DEGs more significantly than LDR exposure. Significant DEGs were subsequently identified in volcano plots with TMM‐normalised values. In the breeding season, *Lsp1*, *Pmel*, *Umodl1*, *Mapre2*, and *C1qtnf1* expression was upregulated, whereas *Ptprk*, *Tspear*, *Arhgap28*, and RP23‐220C16 were downregulated in the contaminated group as compared to the control group. In the non‐breeding season, *Fmo2* and the highly similar *Fmo2* were upregulated in the contaminated group. Out of the DEGs identified, five were considered as potentially significant biomarkers (*Lsp1*, *Ptprk*, *Tspear*, *Fmo2*, and *Fmo2 HS*), and these were further confirmed with qPCR.

Cao *et al*. [35] demonstrated that *Lsp1* expression was directly correlated with immunosuppressive cells, such as M2 macrophages, neutrophils, and regulatory T cells. Moreover, enhanced immunosuppressive gene expression, such as that of programmed cell death 1 and leukocyte‐associated immunoglobulin‐like receptor 1, was observed in macrophages after *Lsp1* upregulation, with *Lsp1* also promoting macrophage migration. These results suggest that *Lsp1* could contribute to the immunosuppressive microenvironment. Furthermore, *Lsp1* is involved in immune checkpoint regulation, and its expression is elevated in the testes of mice exposed to chronic low‐dose‐rate radiation, suggesting a potential impact on acquired immunity [36]. *Ptprk* has been implicated in the regulation of tumourigenesis and proliferation [37]. *Ptprk* downregulation has been reported to stimulate cell proliferation *in vitro*. Moreover, *Ptprk* is closely associated with spermatogonia stem cell activity, particularly signal transduction [38]. *Tspear* knockdown results in the altered expression of genes regulated by Notch, affecting murine hair and tooth development [39]. Lastly, *Fmo2* enhances the release of reactive oxygen species (ROS), particularly of H_2_O_2_ [40]. Morimoto *et al*. [41] revealed that ROS generation is required for stem cell self‐renewal during spermatogenesis in spermatogonial stem cells. In conclusion, our findings suggest that *Lsp1* upregulation enhances the expression of immune‐related genes in the testes and prevents spermatogenic cell apoptosis caused by chronic LDR exposure in large Japanese field mice during the breeding season. Downregulation of *Ptprk* expression is thought to facilitate signalling in spermatogonial stem cells, stimulating their proliferation. Furthermore, *Tspear* plays a critical, previously unrecognised role in sperm morphogenesis by regulating the Notch signalling pathway. *Fmo2* upregulation in non‐breeding contaminated mice promotes spermatogonial stem cell renewal by increasing ROS production. qPCR analysis results were consistent with transcriptome sequencing, confirming the differential expression of *Lsp1* and *Ptprk* in the testes of Fukushima mice. As discussed earlier in our introduction, several laboratory‐controlled mouse experiments have shown that there may exist a threshold dose rate or total dose which could affect spermatogenesis and reproductive function. Our results suggest that exposure beyond this threshold might affect the self‐renewal and differentiation of spermatogonia stem cells in mouse testes. The differences in expression of the selected biomarkers imply enhanced spermatogenesis occurring in large Japanese field mice living in the area affected by the FDNPP accident during both the breeding and non‐breeding seasons.

The current study does have some limitations. Control mice showed a higher body weight than contaminated mice, indicating an older age. Studies have shown that testes transcriptome profile changes are considerably affected by age [42, 43]. There could also be possible transgenerational effects with respect to LDR exposure. The ex‐evacuation zone was contaminated in March 2011, and a few generations could have passed since our collection period in 2012–2013. As mentioned earlier, mice could also have a large area of habitation, which affects the total exposure dose. The transcriptomic profile could also be affected within the short time period of a few hours from capture to euthanasia. While these are all valid and possible factors that could affect our experimental results, our study focuses on wild mice and is not a laboratory‐controlled experiment. It is also hard to repeat the experiment with larger mice exposed to the same environmental conditions during 2012–2013 as the ex‐evacuated zone has been partially decontaminated.

In addition, DEG and other statistical analyses assume that all conditions other than the radiation exposure are identical in the mice. An inherent limitation in all wild animal studies is that gene expression can vary according to many environmental factors (e.g. food availability, temperature, and health conditions). Thus, the control site in Niigata might not represent a ‘true’ control site. To address this, we can conclude that, while significant differences were observed in the two potential biomarkers, there is no definitive evidence showing a causal association between gene expression and chronic LDR exposure.

## Conclusions

This study identified potential germline‐specific radiosensitivity biomarkers in the testes of large Japanese field mice chronically exposed to LDR radiation after the FDNPP accident, in both breeding and non‐breeding seasons. Our study demonstrated that chronic LDR radiation exposure could influence specific genes, highlighting their potential as radiosensitive biomarkers. Our findings provide novel insights into reproductive biology under environmental radiation, and these gene biomarkers are expected to be useful for analysing the effects of chronic LDR radiation exposure on the reproductive system of wild mice. However, although the differences in gene expression found may suggest potential biomarkers, they in no way prove a causal relationship between biomarkers and the response to radiation. It should be noted that radiation is not the only causal factor contributing to the observed changes seen as differences in various health factors could also contribute to differential gene expression.

## Conflict of interest

The authors declare no conflict of interest.

### Peer review

The peer review history for this article is available at https://www.webofscience.com/api/gateway/wos/peer‐review/10.1002/2211‐5463.13927.

## Author contributions

Conceived and designed the experiments: MF, TM, HY. Performed the experiments: ST, IU, AN, HY. Analysed the data: ST, TS, AN, HY. Contributed reagents/materials/analysis tools: RN, YF, VSTG, DA, YS, HS, JG, HI, JS, YK, MGP, AMH, AN. Wrote the paper: ST, HY. English language editing: VSTG, DA.

## Supporting information


**Table S1.** Testis gene expression profiles of the Niigata and Fukushima groups of large Japanese field mice testis during the breeding season.

**Table S2.** Testis gene expression profiles of the Niigata and Fukushima groups of large Japanese field mice during the non‐breeding season.

## Data Availability

The data underlying this article will be shared upon reasonable request to the corresponding author. FASTQ data files of each sample that were obtained in this study were deposited in the DDBJ Sequence Read Archive (DRA) with the following accession numbers: DRR609345–DRR609356.
